# Lithium‐Ion Battery Cathode Recycling through a Closed‐Loop Process Using a Choline Chloride‐Ethylene Glycol‐Based Deep‐Eutectic Solvent in the Presence of Acid

**DOI:** 10.1002/open.202300061

**Published:** 2023-07-26

**Authors:** Delphine Yetim, Lenka Svecova, Jean‐Claude Leprêtre

**Affiliations:** ^1^ Univ. Grenoble Alpes Univ. Savoie Mont Blanc CNRS Grenoble INP, LEPMI 38000 Grenoble France; ^2^ Agence de l'environnement et de la Maîtrise de l'Energie 49004 Angers Cedex 01 20 Avenue du Grésillé B. P. 90406 France

**Keywords:** closed-loop process, cobalt recovery, deep eutectic solvent, LIBs leaching, lithium recovery

## Abstract

This study evaluates the ability of a choline chloride:ethylene glycol‐based deep eutectic solvent (DES) to dissolve lithium cobalt oxide (LCO) which is used as a cathode active material in Li‐ion batteries. Both a commercial powder and spent cathodes have been used. It was demonstrated that if HCl is added in a small proportion, a rapid and efficient LCO dissolution can be achieved. Indeed, if more than three protons are added per one cobalt atom present in the LCO structure, a complete dissolution of the material is accomplished within 2 h at 80 °C. This result might be considered as a viable alternative compared to the literature where much longer reaction times and higher temperatures are applied to achieve similar results with the same DES system used either pure or in presence of additional reducing agents. It was further demonstrated that Co and Li can be fully precipitated after Li_2_CO_3_ addition. This precipitation does neither pollute the DES nor leads to its degradation provided the pH does not exceed 10. Finally, it was shown that two additional reuse cycles can be carried out without any decrease of recovery efficiency, while no degradation products have been detected within the DES phase.

## Graphical abstract

A novel closed‐loop method for Li and Co recovery from a lithium cobalt oxide (LCO) material using a deep eutectic solvent (DES) based on choline chloride (ChCl) and ethylene glycol (EG) with an additional source of protons. This process allowed to quantitatively recover Co and Li, highlighting the importance of adding a precise amount of protons to drive Co dissolution. In addition, the DES was used in three successive leaching/recovery cycles without any degradation.

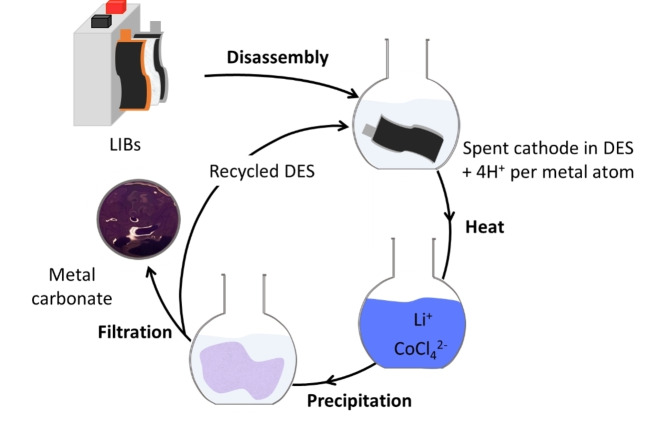

## Introduction

### Lithium‐ion batteries recycling overview

The recycling of spent lithium‐ion batteries (LIBs) has attracted a global attention in recent years due to the overexploitation of critical resources (e. g., Co, Li), their polluting mineral extraction methods, their unequal distribution in the earth crust and the complicated end‐of‐life management of this toxic waste.^[1]^ To face these issues, the European Union directive draft published in December 2020,^[2]^ proposes to increase the mandatory LIBs recycling rate from 50 to 65 % and to use at least 12 % of recycled cobalt for the production of new batteries from 2025. To achieve these goals, two main alternatives are available for spent LIBs recycling, that is, pyrometallurgy and hydrometallurgy. Each of them exhibits its own advantages and disadvantages. Furthermore, while they are generally presented as two different recycling paths, they are often combined at the industrial level.^[3]^ Pyrometallurgy is a thermal treatment of the batteries at very high temperatures ranging from 600−1000 °C^[4]^ leading to a mixed alloy containing a large part of the metals (Co, Cu etc.) and a slag. This method is very energy‐intensive and generates toxic gases (e. g., HF originating from the decomposition of the LiPF_6_ electrolyte and the PVDF binder) and dust. It leads also to a loss of materials as Li and Al are generally lost in the slag. Hydrometallurgy is less prohibitive in terms of energy costs, due to the fact that the process is carried out at lower temperatures (40−100 °C). The principle of this chemical approach is to dissolve metals in their ionic form while targeting a high dissolution efficiency and, if possible, a certain selectivity.

### Focus on hydrometallurgy approach

In conventional hydrometallurgy, strong mineral acids are traditionally used for the dissolution of the most common oxide‐based LIBs standard cathode materials (LCO ‐ LiCoO_2_, NMC ‐ LiN_x_Mn_y_Co_z_O_2_, NCA ‐ LiNiCoAlO_2_, LMO ‐ LiMnO_2_), while moderate heating (60−90 °C) is often applied as shown in Table S1. This table does not claim to be exhaustive due to the plethora of articles on this topic, however, the selected results well display the achieved efficiencies and the applied experimental conditions.

Regarding the LCO material and taking into account the oxidation state of the present cobalt (i. e., Co^III^) and its low solubility, its dissolution must be accompanied by Co^III^ reduction into Co^II^. This is either achieved in H_2_SO_4_ media by the action of H_2_O_2_ according to the Equation 1:^[5]^

(1) $$2LiCoO_{2}+H_{2}O_{2}{+3H}_{2}{SO}_{4}↔{Li}_{2}{SO}_{4}+2CoSO_{4}+4H_{2}O+O_{2}$$

Or, upon HCl addition, the chloride ions brought by the acid are oxidized to chlorine gas while reducing the cobalt according to the Equation (2) considering the higher E° of the Co^2+^/Co^3+^ couple compared to the Cl^−^/Cl_2_ one (i. e. 1.83 V vs 1.36 V vs. ESH, respectively):

(2) $$LiCoO_{2}+4\;HCl↔{Li}^{+}+{Co}^{2+}+\frac{1}{2}{\;Cl}_{2}+3\;{Cl}^{-}+2{\;H}_{2}O$$

Under these experimental conditions, the relatively short reaction times (from 30 min to 6 h) leads to quite efficient dissolution rates (Table S1). Moreover, even if the leaching is optimized for the Co recovery, it is also accompanied by the quantitative Li dissolution.

However, these treatments require quite aggressive media, unavoidably leading to the production of acidic effluents. Organic acids alone or in association with H_2_O_2_ might be an interesting alternative (short reaction times at low temperature), combining the action of protons with the necessary redox reactions and metals complexation helped by its association with the ligand anions from the acid species.^[16]^

### Deep eutectic solvents as alternative leaching media

Another interesting option that has recently started to attract the attention of scientists are the so‐called deep eutectic solvents (DESs). First described by Abbott et al. in 2001,^[17]^ the DESs have quickly shown interesting properties for many applications including the metals recycling.^[18]^ Although the exact definition is still under debate, it is generally accepted that the DESs are homogeneous systems composed of a hydrogen bond donor (HBD) and a hydrogen bond acceptor (HBA); the mixture of both components gives, at a particular proportion, a liquid with melting point well lower with respect to the individual melting points of both components. These mixtures are easy to prepare by their simple mixing under a moderate heating. Many of them are liquid at room temperature, although some of them exhibit high viscosities. A selection of DESs used for LIBs active materials leaching is summarized in Table 1.

**Table 1:** Non‐exhaustive list of DES based leaching agents used in the literature and the corresponding experimental conditions and the achieved leaching efficiencies (ChCl=choline chloride, EG=ethylene glycol, PEG=polyethylene glycol, PSA=p‐toluenesulfonic acid).

Cathode active material	Leaching agent	Active material/ DES mass ratio	Temperature [°C]	Time	Efficiency	Ref.
LCO	ChCl : EG (1 : 2)	0.02 g⋅g^−1^	180	24 h	70 % Co	[6]
NMC	ChCl : EG (1 : 2)	0.02 g⋅g^−1^	160	20 h	90 % Co, 10 % Ni	[7]
LCO	ChCl : Citric acid (CA)+35 % H_2_O (1 : 1)	20 g⋅L^−1^	40	60 min	>98 % Co	[8]
LCO	ChCl : Oxalic acid (OA) (1 : 1)	0.007 g⋅g^−1^	180	10 s	Quantitative	[9]
LCO	PEG : Ascorbic acid (AA) (6 : 1)	0.01 g⋅g^−1^	80	72 h	84.2 % Co	[10]
LCO	ChCl : Urea (1 : 2)	0.02 g⋅g^−1^	180	12 h	95 % Co, 95 % Li	[11]
LMO, LNM, LNC	ChCl : Lactic acid (2 : 1)	0.1 g⋅g^−1^	70–105	10–15 h	100 %	[12]
LCO	Guanidine hydrochloride : Lactic acid (1 : 2)	Not indicated	80	24 h	Not given	[13]
LCO	ChCl : Succinic acid : EG (1 : 1 : 1)	0.003 g⋅g^−1^	140	16 h	>95 % Co	[14]
LCO	PEG : PSA (1 : 1)	0.02 g⋅g^−1^	100	24 h	99.5 % Co	[15]

The majority of the identified studies involves choline chloride as the HBA, while ethylene glycol, organic acids or urea are used as HBDs. As an example, Tran et al.^[6]^ have demonstrated that when using a ChCl:EG mixture, long reaction times and high temperature are required to achieve an acceptable Li and Co dissolution. A dissolution efficiency of 95 % from LiCoO_2_ commercial powder was only obtained when 220 °C was maintained during 24 h. If a lower temperature was used (180 °C), reaction times of up to 72 h were necessary. Wang et al.^[11]^ obtained a Li and Co leaching efficiency of 95 % at 180 °C with a reaction time of 12 h using a ChCl:urea mixture, whereas Peeters et al.^[19]^ achieved the dissolution of LCO in the presence of Al and Cu in a DES composed of ChCl and citric acid in 60 minutes while heating to 40 °C. However, to reach this relative shorter leaching time, the authors added 35 % wt. of water into their system to reduce its viscosity. Li et al.^[9]^ have used another system containing an organic acid (ChCl:oxalic acid), achieving an immediate dissolution within a few seconds when working at 180 °C, accompanied by a significant production of bubbles. However, when lowering the temperature to 90 °C, more than 2 h were required to observe a complete dissolution. Thus, regarding Table 1, the leaching efficiency seems to be dependent on the DES components, the reaction time and the temperature.

### DESs leaching mechanism and their stability

In this context, after having reported the DESs’ ability to dissolve metal oxides,^[20]^ the Abbott group has investigated the influence their “pH” and their hydrogen bond donor ability on their ability to dissolve the metal oxides.^[21]^ In this work, the solubilities of several oxides in different DES systems involving choline chloride as one of the constituents have been compared at 50 °C and 48 h. The dissolution was in general low in DESs with neutral pH and poorly acidic HBD (EG, urea, glycine) compared to systems with lower pH and/or containing an acidic HBD (such as oxalic acid). However, it was observed that the complexing ability of the conjugate base from the investigated acids seemed to be the key point to the oxides’ dissolution. It was further proposed that the dissolution proceeds via a three‐step mechanism starting with the oxide protonation creating positively charged sites attracting negatively charged species to the solid surface. Then, the intermediate species are believed to form complexes through ligand exchange with Cl^−^ after M−O bond cleavage. Under these conditions, the chlorometallate species, such as CoCl_4_^2−^, have been identified in the leachates as main species for all metals with the exception of nickel.^[21]^ It seems thus that both DES components impact the dissolution process; the HBDs in a more relevant role for the dissolution itself, while the HBA (here ChCl) contributes the complexing agent that enhances the dissolution of species in solution by formation of chlorido complexes. Moreover, an additional reduction step is necessary in the case of some oxides (MnO_2_, Fe_3_O_4_ and Co_3_O_4_). As observed by Pateli et al.,^[21]^ the solubilities of oxides featuring a metal center with a lower oxidation state were systematically higher than those with the same metal in a higher oxidation state as observed in the comparison between CoO and Co_3_O_4_. Metal speciation in the solid matrix is thus a critical aspect because it will determine how quickly the metal oxide will be dissolved. Additionally, it has to be emphasized that the dissolution rates at 50 °C seem to be, in general, quite low in spite of the rather low viscosity of the studied systems.^[21]^ As shown in Table 1, the presence of organic acids in the DES appears to have a positive effect on the dissolution kinetics, otherwise a very high temperature or a presence of an additional reduction agent are necessary to achieve acceptable efficiencies.^[8]^ The LCO dissolution in a pure ChCl:EG system is efficient only if long times and high temperature are combined.^[6]^

In the absence of an additional reducer, the DES (or one of its components) must play the reducer role. However, there is no consensus in the literature regarding which of the ChCl:EG components is responsible. Tran et al. have hypothesized that the EG plays this role as it is the case in the polyol synthesis of noble metallic nano‐catalysts (at high temperature >180 °C).^[6]^ However, Peeters et al.^[19]^ have focused on the thermal degradation of ChCl:EG mixture and have demonstrated that it is not stable at 180 °C, limiting its recyclability while producing some toxic reaction products such as trimethylamine and 2‐chloroethanol. It was further suggested that these side products could originate from the cobalt(III) reduction by the ChCl (and not the EG) which simultaneously undergoes a radical β‐hydrogen abstraction reaction. Moreover, similar decomposition products have also been identified by Schiavi et al.^[7]^ In their following work, Schiavi et al.^[22]^ have furthermore demonstrated that the degradation products might be profitable for the leaching process, while, in contrast, they could also compromise the reuse of the DES after the metal extraction step.

### Metals recovery from the DESs

Once the metals are dissolved, they must be recovered in a solid form, either as salts, hydroxides or in their metallic form. In conventional hydrometallurgy, the separation and purification techniques (using solvent extraction and ion exchange) are combined to the recovery operations (precipitation, crystallization and electro‐deposition).^[23]^ It is important to keep in mind that the precipitation is most often carried out by the acid neutralization with a base, thus leading to its loss, preventing any further direct reuse of this agent. A similar approach has been adopted within the DES media by Schiavi et al.,^[7]^ who performed a solvent extraction using D2EHPA (di‐(2‐ethylhexyl)phosphoric acid) to isolate the cobalt dissolved in a DES, prior to its recovery by precipitation induced by oxalic acid addition as cobalt oxalate. Tran et al.^[6]^ have demonstrated both the feasibility of direct precipitation by Na_2_CO_3_ addition and Co electrodeposition without their optimization, while Wang et al.^[23]^ have tested several precipitation agents (Na_2_CO_3_, NaOH, H_2_C_2_O_4_).

### Choice of the DES leaching medium

In this context, our objective was to develop an efficient LCO recovery process based on the use of a DES. This process should be as simple as possible, using a minimum of chemical products, while being rapid and if possible carried out below 100 °C. Regarding the DES choice, based on the state‐of‐the‐art conclusions, the presence of acid is required in order to reach relative short reaction times at low temperature (<100 °C). However, organic‐acid‐based DESs are characterized by high viscosities, hindering the mass transfer, extending the reaction times and also complicating all pouring and separation operations (Table S2). Moreover, they are also known to undergo some degradations. This is well described by Rodriguez Rodriguez et al.^[24]^ in the case of carboxylic acid:ChCl DESs, which exhibit some degradation leading to the corresponding esters.

We have thus been inclined to choose the ChCl:EG based DES due to its excellent physico‐chemical properties (low viscosity, low melting point) and we have tried to find a way to decrease the reaction time and the temperature of this operation. In this context, we focused on a DES involving a strong free acid. However, the use of trifluoromethanesulfonic acid (TFSA), as proposed by Pateli et al.,^[21]^ was excluded, because this acid is too expensive and might additionally pollute the DES. Thus, we have investigated ChCl:EG DES with an additional cost‐efficient acid, namely HCl, that contributes the necessary protons to assist in the cleavage of the metal‐oxygen bond as proposed by Pateli et al..^[21]^ In parallel, it does not pollute the DES since chloride ions are already present (choline chloride). The objective is to take advantage of the synergy of both the DES and the dopant HCl to achieve acceptable leaching yields at relatively low temperatures and short reaction times. Thus, it is expected that the added acid will contribute to the acceleration of the oxide dissolution without impacting or even improving the complexation phenomenon and the recyclability of the DES. Finally, the feasibility of the dissolved metals recovery by precipitation and the DES reusability will be described in this paper.

## Results and Discussion

### ChCl:EG with an additional protons’ source as leaching agent for efficient LCO dissolution

Prior to checking the DES ability to dissolve the LCO material, we focused on the determination of the maximum working temperature to carry out the leaching without degrading the DES. These preliminary tests were carried out with the DES mixed with HCl solution, whose final concentration within the DES was arbitrarily fixed at 0.8 mol⋅L^−1^. The prepared system was then heated to an arbitrarily chosen temperature of 87.5 °C for 2 h to check the DES stability and to estimate the contribution of the DES thermal degradation during the dissolution process. The DES was analyzed before and after the experiment and the corresponding NMR spectra of both mixtures are shown in Figure S1 (Supporting Information).

When comparing the signal at 3.2 ppm (4 CH_3_ groups of ChCl) and the one at 3.7 ppm (two CH_2_ from EG) in Figure S1, their integral ratio (i. e. 3) indicates that there was no evolution in the DES composition. In parallel, knowing that some authors observed DES degradation at high temperature leading to some volatile species,^[24]^ we used the ethyl‐group‐associated resonance of TEAB (tetrafluoroborate) in the insert to check any evolution of the integral of the EG and the ChCl peaks. The amount of the two components remains unchanged, showing that, under these experimental conditions, the DES thermal degradation in the presence of HCl can be considered as negligible over, at least, two hours.

**Figure 1:**
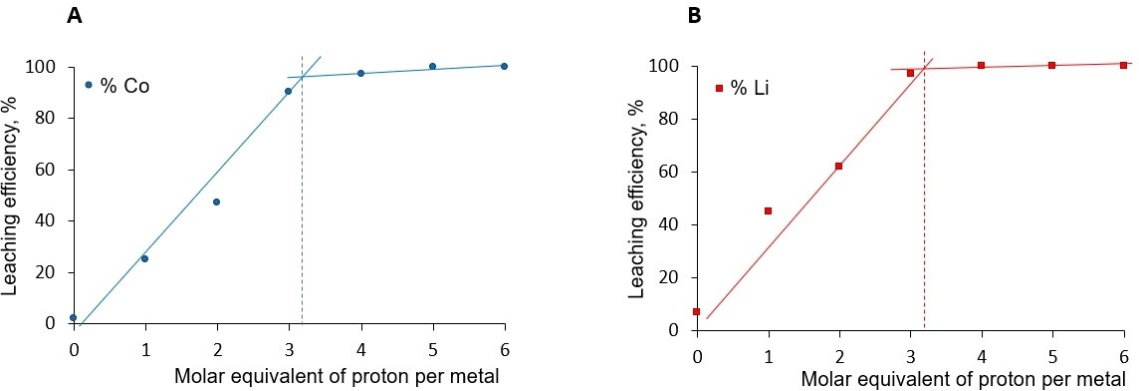
Evolution of the dissolution efficiency of commercial LCO powder as a function of HCl molar equivalents quantity added per mole of LCO, A) Co (blue circles) and B) Li (orange squares). Each experiment has been carried out at 87.5 °C during 2 h with powder/DES ratio of 1 : 50 wt. and the concentration was determined by AAS (solid lines in both figures serve as visual guides only).

Once the DES stability was verified, the LCO dissolution efficiency was studied by varying the H^+^/Co molar ratio (Figure 1). This ratio ranges from 0 to 6 molar equivalents of protons per mole of Co. Taking into account the fact that the solid/liquid ratio was kept fixed at 1 : 50 wt., this corresponds to an effective concentration of acid within the DES ranging from 0 mol⋅L^−1^ to 1 mol⋅L^−1^.

In the absence of additional protons, the Li dissolution is close to 7 %, while very low Co co‐leaching is noticed (i. e., 2 %). The non‐negligible Li dissolution is presumably related to the lower Li interaction within the LCO structure as illustrated by its conductivity (mobility) in the material, allowing its easier dissolution.^[25]^ However, its higher dissolution might also be attributed to the presence of some excess lithium in the powder (Co : Li ratio 0.98 : 1, determined experimentally, not shown here). As detailed in the Introduction section, the cobalt dissolution requires the previous cleavage of the Co−O bond within the LCO structure whose robustness does not allow significant Co dissolution without a protons’ addition under the tested experimental conditions (2 h, 87.5 °C). Although much attention is traditionally given to the role of acid in this dissolution process, the role of water present in the DES is somehow overlooked. Indeed, the literature frequently suggests that the dissolution of this oxide is driven by the protons provided by the acid only. However, most of the articles do not consider the water presence in DESs (which are not dried, though), whereas without any proton source, the Co^III^ reactivity towards water could lead to a protons release [Equation (3)] as suggested in the literature in the case of the use of cobalt oxide particles as a water oxidation catalyst.^[26]^

(3) $${2Co}^{3+}+H_{2}O\to {2Co}^{2+}+\frac{1}{2}O_{2}+{2H}^{+}\;$$

As shown in Figure 1, lithium and cobalt dissolution increase regularly after the HCl addition to quantitative levels at approx. 3 molar equivalents of protons per LCO, levelling off beyond this ratio. This unambiguously demonstrates the positive effect of protons’ addition that helps to weaken the Co/O interaction and to break the LCO crystalline structure according to Equation 4:

(4) $${2LiCoO}_{2}+{6H}^{+}\to \;{2Co}^{2+}+{2Li}^{+}+{3H}_{2}O+\frac{1}{2}O_{2}$$

It has to be highlighted that the proton sources originate probably both from the added HCl and the H^+^ released by the reaction of Co^3+^ (E°=1.83 V vs. SHE) and H_2_O according to Equation (3). Indeed, according to the mechanism proposed by Pateli et al.,^[21]^ four protons are necessary to fully protonate both oxygen sites present in the LCO unit, however, one proton will then be provided by the water oxidation by Co^3+^.

The data in Figure 1 thus fit the expected amount required for quantitative Co and Li dissolution well, since more than 90 % efficiency is reached when three protons per present Co are added. This result is sustained by the DES pH measurements after two hours of the reaction (pH measured after DES dilution in water and recalculated by taking account into the dilution factor). For experiments from 0 to 3 molar equivalents of HCl per mole of Co, the final pH was close to 4−5, testifying to the total consumption of the acid. On the other hand, for higher HCl amounts, the final pH values approached pH 1, demonstrating that the added protons are in excess.

**Figure 2:**
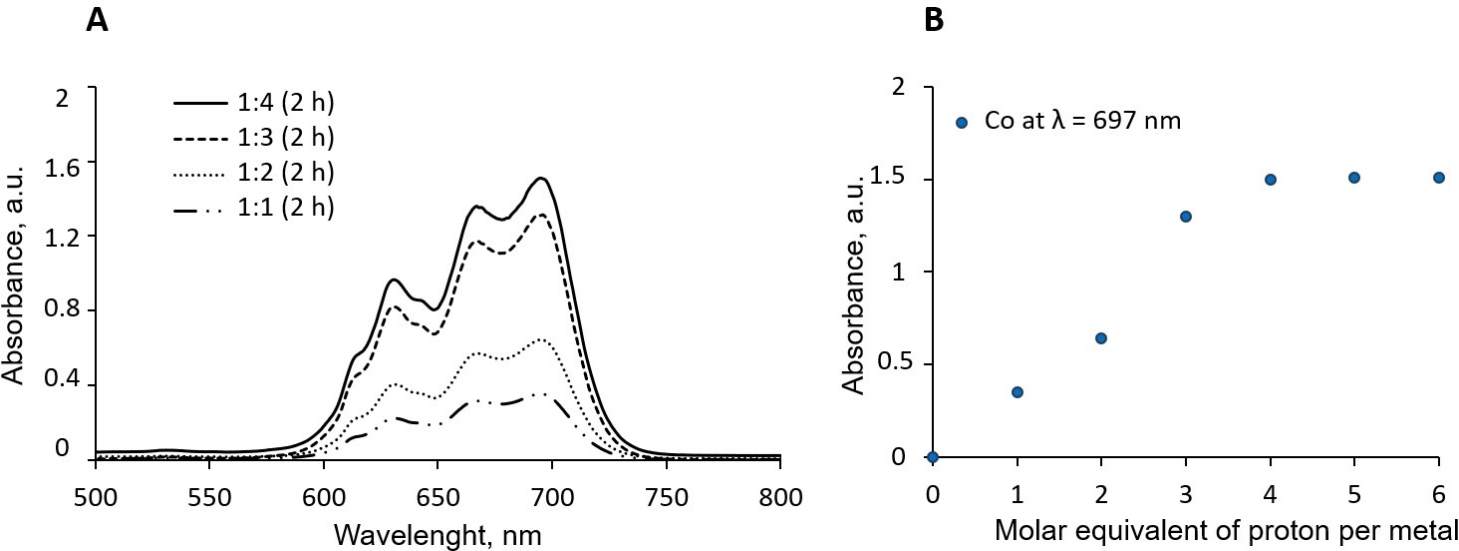
(A) UV‐visible spectra of LCO dissolution in DES containing 1, 2, 3 and 4 molar equivalents of HCl added per mole of cobalt at 87.5 °C during 2 h with LCO/DES ratio of 1/50 wt.. (B) Evolution of absorbance at λ=697 nm as a function of the number of added protons in DES ChCl:EG. A quartz glass cuvette (d=0.1 mm) has been used.

The efficiency of the Co dissolution can also be easily monitored using UV‐visible spectroscopy. After 2 h of reaction at 87.5 °C in the presence of 1, 2, 3 and 4 molar equivalents of HCl, the UV‐visible spectra (Figure 2, A) attest, as expected, that the Co dissolution leads to CoCl_4_^2−^ complex formation,^[27]^ which is in accordance with the observation of Pateli et al.^[21]^

In addition, the evolution of the absorbance at 697 nm, characteristic of the CoCl_4_^2−^ species, as a function of the amount of added protons (Figure 2, B), follows exactly the same trend as the Co dissolution curves determined by AAS (Figure 1, A), testifying to the formation of CoCl_4_^2−^ as the main species present over the whole range of tested experimental conditions.

Although detecting the same final species, i. e., CoCl_4_^2−^, Schiavi et al.^[22]^ have proposed a different, and very complex, leaching mechanism for Co dissolution within a pure ChCl:EG mixture (at 180 °C) proceeding via the production of Cl_2_ by the oxidation of ChCl, that would immediately combine with Cl^−^ ions by creating the trichloride ion, Cl_3_^−^, detected at 247 nm on the UV‐vis spectrum of a thermally aged ChCl:EG sample. According to the same authors, in the presence of significant quantities of Cl_2_, a band at 362 nm might be detectable, too. Moreover, the Cl_3_^−^ ion then plays a dominant role in metallic oxide dissolution via the oxygen oxidation into the superoxide ion, O_2_^−^, detectable at approx. 250–270 nm, but was not really detected by the authors due the overlay of signals of several species in the same region. The “liberated” metallic cations can then easily complex present chloride ions. The reduction of Co^III^ might, according to Schiavi et al.,^[22]^ happen during Cl^−^ oxidation into Cl_2_, but the role of organic molecules originated from ChCl degradation cannot be excluded.

As shown in Figure S2, none of the mentioned species (Cl_2_, Cl_3_^−^ and O_2_^−^) were present in our system as evidenced by the absence of corresponding absorption bands from the recorded UV‐Vis spectrum. Moreover, in the case of the study by Schiavi et al.,^[22]^ many different degradation products have been identified by GC‐MS and NMR spectroscopy, some of them probably involved, in a positive manner, in the active material dissolution. None of these species have been detected in our system as will be described in the section “Metals recovery and DES reuse”.

**Figure 3:**
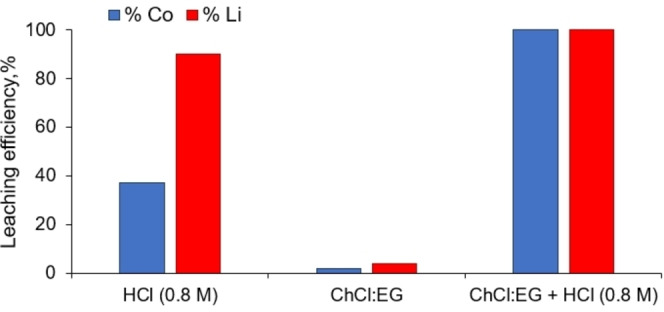
Comparison of leaching efficiency of the cobalt and lithium from LCO powder in presence of HCl (0.8 M), ChCl:EG and ChCl:EG + HCl (0.8 M), at 87.5 °C during 2 h, with LCO/solvent ratio of 1/50 wt.

Finally, to better understand the advantages of mixing the DES with an additional source of protons, a leaching of LCO powder was carried out in the presence of aqueous HCl (0.8 M) only and is compared in Figure 3 to pure ChCl:EG without any acid addition and ChCl:EG with HCl (0.8 M) at a previously fixed s/l ratio.

As shown in Figure 3, the leaching efficiency of Co obtained for the aqueous HCl and the DES alone are very low (close to 5 % for ChCl:EG and 37 % for HCl at 0.8 M in accordance with the literature for this HCl concentration, temperature and s/l ratio^[28]^) compared to the leaching efficiency obtained in the case of ChCl:EG+HCl, which reaches 100 %. This unambiguously demonstrates the benefits of the HCl and DES synergy towards the leaching efficiency. It was thus decided to carry out all following experiments with the addition of 4 molar equivalents of proton per mole of Co by the addition of HCl in order to have a slight proton excess within the leaching medium.

In addition, several works have shed light on the influence of chloride concentration on the dissolution process.^[29]^ In our study, it was demonstrated that working in a chloride medium (ChCl) promotes the formation of CoCl_4_^2−^ species due to the relatively low water concentration (compared to the aqueous media) and the strong interactions between the metal and the ligand. A comparison of UV‐Vis spectra is given in Figure S4, comparing the spectra of a solution of cobalt in HCl and DES media showing that, for the same concentration of cobalt, the absorbance of a band corresponding to the CoCl_4_^2−^ species is ten times higher in the DES (4.25 M of chloride) compared to the aqueous solution of HCl containing 8 M of chloride.

To conclude, the acidity of the media plays an important role in the dissolution efficiency as it allows to break the oxide structure and to facilitate its dissolution. The presence of the DES, and namely the chloride ligand, allows then to complex and to stabilize the dissolved metals in solution.

### Influence of temperature and time on the DES leaching efficiency of the LCO powder

**Figure 4:**
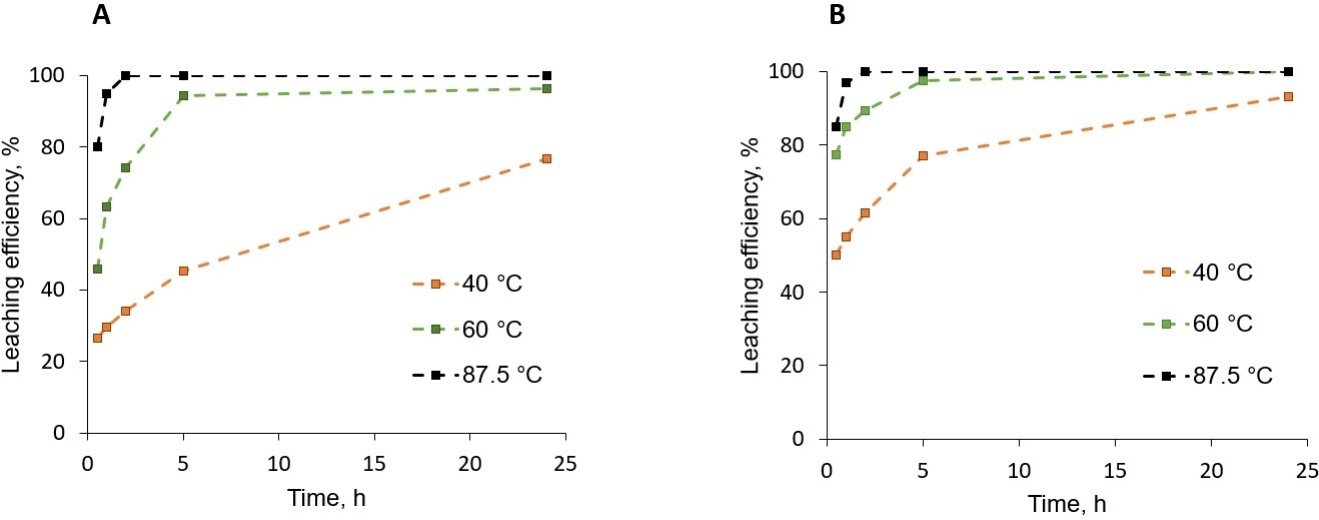
Influence of temperature on cobalt (A) and lithium (B) leaching efficiency from LCO commercial powder using ChCl:EG (1: 2) deep eutectic solvent at 40 °C, 60 °C and 87.5 °C with a an active material/solvent ratio of 1/50 wt., and addition of 0.8 M of HCl (4 molar equivalents per metal).

Furthermore, the influence of DES temperature on the LCO dissolution efficiency was investigated (Figure 4) and, as expected, at rather low temperature (i. e., 40 °C) and in the presence of 4 molar equivalents of HCl, the Co and Li dissolution is not quantitative for the same reaction time (2 h). The dissolution kinetics are more rapid at 60 °C, but the dissolution is not yet fully quantitative and the temperature needs to be increased up to 87.5 °C to reach a quantitative LCO dissolution in the same reaction time. Interestingly, the lithium dissolution seems to be more rapid and efficient compared to cobalt namely at low temperature confirming our previous observations.

Compared to Tran et al. who achieved a quantitative dissolution in the ChCl:EG system at 180 °C and 72 h,^[6]^ it has to be highlighted that much shorter time and lower temperature are necessary in our conditions, confirming the beneficial effect of proton source addition. The same conclusion can be drawn regarding the results obtained by Schiavi et al.^[7]^ who have shown that even in the presence of Cu and Al reducers, 160 °C and 20 h are necessary to achieve a complete dissolution in the same DES. On the other hand, comparable results can be found in the literature for acid‐based DESs.[^8^, ^9^] However, as previously mentioned, this kind of DESs based on carboxylic acid and ChCl are subject to degradations (esterification) during the leaching process.^[24]^

### Comparison of the leaching kinetics of a spent LCO cathode and the commercial LCO powder

**Figure 5:**
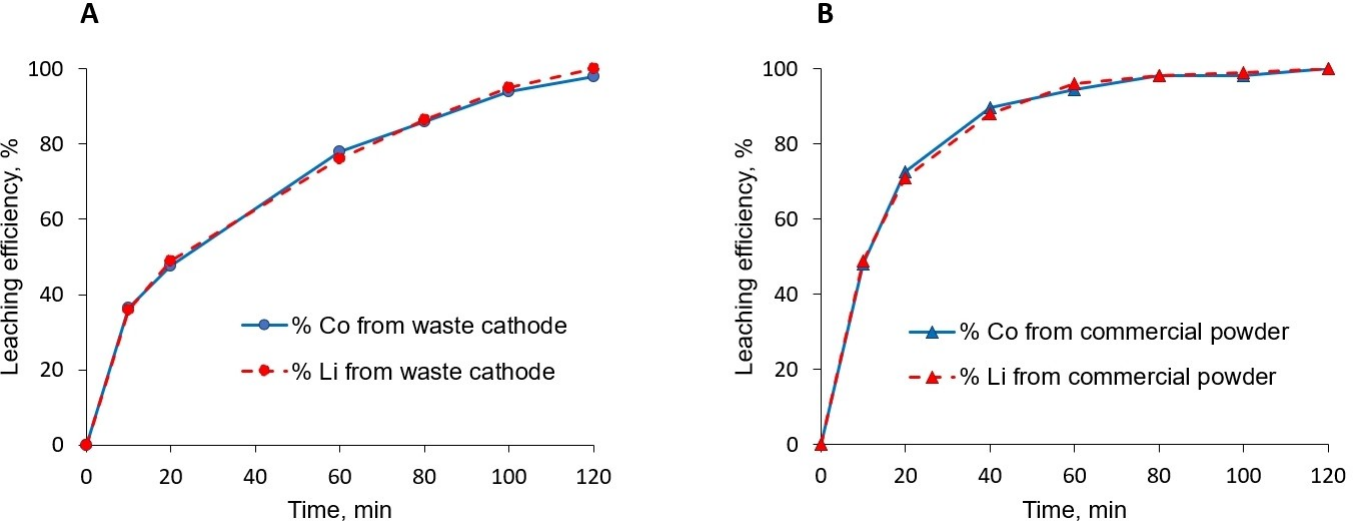
Leaching efficiency of the spent LCO cathode (A) and the LCO commercial powder (B) using ChCl:EG (1 : 2) mixture at 87.5 °C with a solid/liquid ratio of 1 : 50 wt. and addition of 4 HCl equivalents per Co atom (corresponding to 0.8 M of HCl within the leaching medium).

When performing a conventional LIBs recycling at an industrial scale, the so‐called black mass (active materials’ concentrate) issued from the spent Li‐ion batteries pretreatment may contain a certain amount of Al originated from an incomplete separation of the active material from its current collector. The presence of aluminum could then contribute to the consumption of protons, through a parasitic corrosion/dissolution reaction leading to the production of H_2_. The influence of Al on the LCO leaching process was investigated by comparing the dissolution kinetics of the LCO powder and an LCO‐based spent cathode (containing Al collector and a binder). Each experiment was carried out with 45.5 cm^2^ of cathode (corresponding to 1 g of active material) and 1 g of LCO powder. The dissolution of the LCO powder (Figure 5, B) and the spent cathode (Figure 5, A) were compared at the previously determined optimum experimental conditions (87.5 °C, solid / DES wt. ratio of 1/50, and 4 molar equivalents of HCl per metal).

Both materials are fully dissolved within 2 h and the presence of the Al current collector does not seem to affect the dissolution efficiency of the spent cathode. The contribution of Al corrosion to H^+^ consumption can be considered negligible. It thus seems that either the kinetic of LCO dissolution is favored over the corrosion phenomenon or that some Al passivation occurs, limiting its reactivity.^[30]^

However, the slopes of both kinetics curves differ, indicating a lower dissolution rate of the spent cathode compared to the LCO powder within the first minutes, which is a surprising phenomenon. The cathode material exhibits, indeed, lower grain size (0.1 μm – 1 μm, determined by SEM observations, not shown) compared to the commercial powder (5–15 μm, determined by SEM observations, not shown) and a faster H^+^‐oxide interaction is thus expected. Nevertheless, the presence of the PDVF binder, which agglomerates the grains and stick them to the Al collector, might restrain the H^+^ access to the active material, thus hindering the dissolution process and explaining the observed difference.

Scanning electron microscope observations performed on the leaching solid residues sampled at 10 min and 40 min (Figure S3) testify to a progressive dissolution of the cobalt oxide only (disappearance of the Co signature) associated to the formation of insoluble Al_2_O_3_. In this way, the corroded aluminum foil was manually removed from the DES after the active material detachment.

### Metals recovery and DES reuse

**Figure 6:**
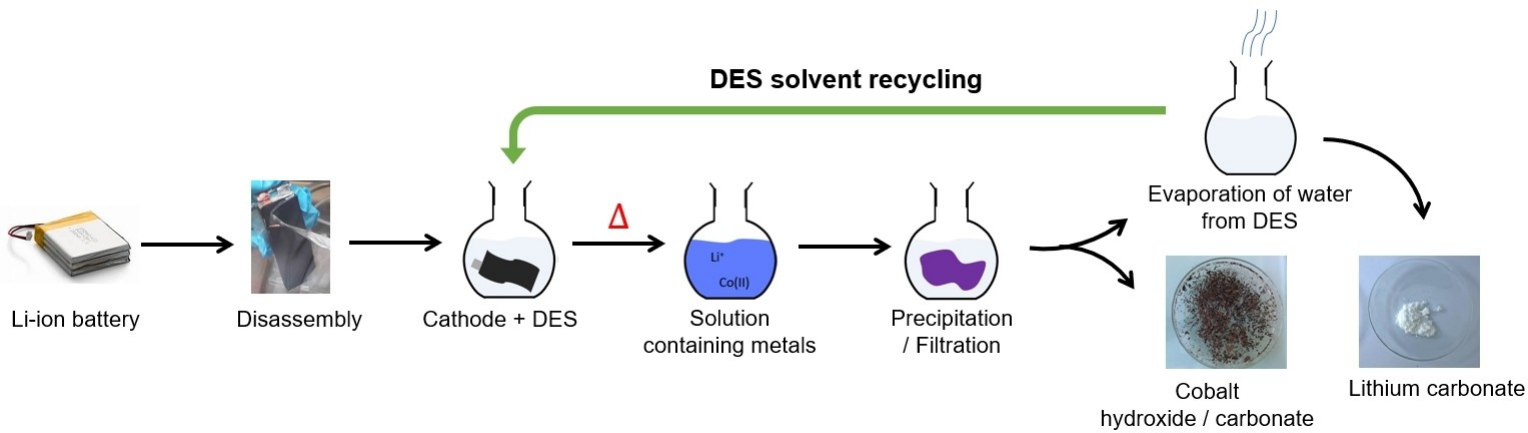
Experimental roadmap of DES recycling in a closed‐loop manner.

Once the dissolution is achieved, the Co recovery either in the form of a hydroxide or as a carbonate has to be performed according to the scheme shown in Figure 6. Li_2_CO_3_ and LiOH were tested as precipitation agents to avoid the pollution by other alkali cations (Na^+^, K^+^) and simplify the DES reuse. It is important to highlight that when LiOH was used as precipitation agent at pH>12, the DES turned yellow and a smell of ammonia was detected. This is an indication that Hoffman's elimination reaction might have occurred at this very basic pH leading to the formation of an enol or an aldehyde (Figure S5).^[31]^ Thus, in order to prevent the DES degradation, we systematically operate at pH<10 using a lithium carbonate solution of 0.2 mol⋅L^−1^. As indicated previously, the final pH after the leaching process is within the pH range of 4–5. Li_2_CO_3_ or LiOH (0.2 mol⋅L^−1^) solution has been added dropwise until the final pH between 10 and 12 has been reached.

Typically, the complete precipitation of the cobalt contained in 50 mL of leachate (0.2 mol⋅L^−1^ of Co) at pH ∼4−5 needs a volume of 100 mL of lithium carbonate (0.2 mol⋅L^−1^) to reach pH 10. No cobalt traces have been detected in the filtrate after the precipitate separation. All Li^+^ ions remained in the filtrate, however they could be either removed by CO_2_ bubbling or by precipitation after the water evaporation. The feasibility of both approaches have been confirmed (results not shown), however, as the water must be removed before the DES reuse, this technique was preferred. Both obtained precipitates were washed with water and dried at 100 °C over two days, resulting in a brown amorphous powder probably corresponding to a mixture of cobalt hydroxide/carbonate (CoCO_3_, Co(OH)_2_) and a white precipitate shown in Figure 6 (photo on the right).

**Figure 7:**
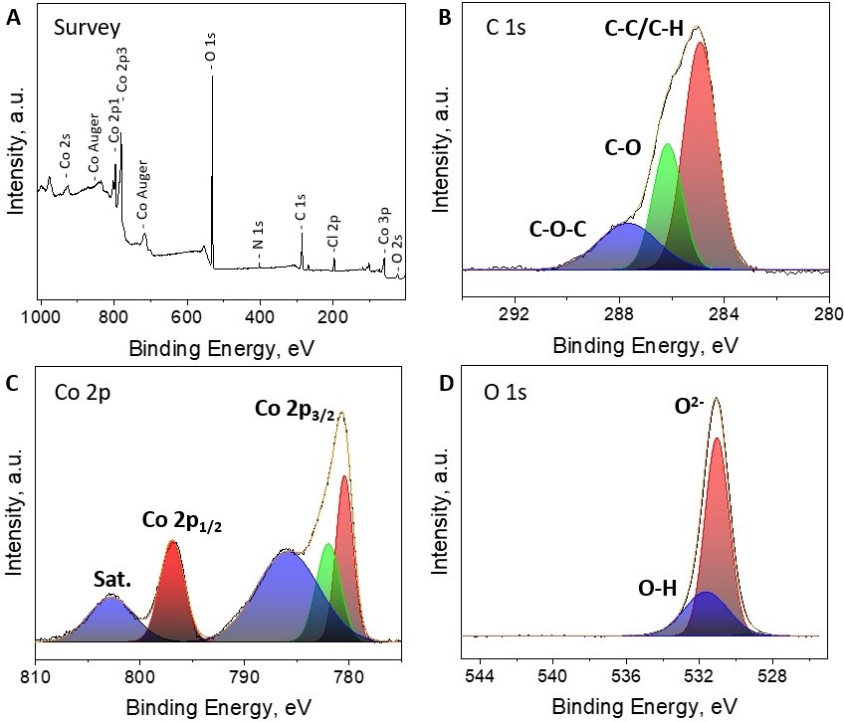
View of (A) the full XPS spectra of Co precipitate and the high‐resolution of C 1s (B), Co 2p (C) and O 1s (D) spectra.

X‐ray diffraction analysis was also performed on the cobalt‐containing precipitate, but being amorphous, it was not possible to obtain a clear diffraction pattern. XPS analysis was therefore performed on the cobalt powder shown in Figure 7. The general survey spectrum (Figure 7, A) reveals the contributions of several elements, that is, cobalt, oxygen, carbon and minor contributions of nitrogen and chlorine. High resolution core‐level spectra have then been recorded for C 1s, Co 2p and O 1s lines. The Co lines (Figure 7, C) corresponding to the BE of 780.4, 782.0 and 786.0 eV accompanied by their satellite peaks confirm the presence of Co in its Co^II^ oxidation state. The O 1s (Figure 7, D) line confirmed the presence of oxygen mainly as a carbonate compound (O^2−^ contribution) with a peak at 531 eV. However, a small contribution of O−H bond is highlighted by the deconvolution of the obtained spectra. Thus, a mixture of a cobalt carbonate with a cobalt hydroxide was probably obtained. As expected, the C 1s line has been detected (Figure 7, A) and is probably at least partially ascribed to the presence of adventitious carbon commonly detected by the XPS technique.^[32]^ However, a partial contribution of DES traces cannot be excluded despite significant rinsing of the precipitate. This hypothesis is strengthened by the detection of N 1s and Cl 2p lines on the survey spectrum with BE of 402 eV and 200 eV respectively. The nitrogen might originate from ChCl traces, while the presence of chlorine might be both explained by ChCl together with some remaining traces of CoCl_4_^2−^ complex. Regarding the C 1s line (Figure 7, B) a strong contribution of organic carbon (C−C/C−H) is detected at 284.9 eV (both DES contamination and adventitious carbon presence), however, the deconvolution reveals the presence of C−O−C (286.2 eV) and O−C=O (287.7 eV) confirming the presence of carbonate. Thus, the obtained precipitate is very probably composed of a mixture of cobalt carbonate and cobalt hydroxide, while the latter seems to be minor. As shown by Riechers at al.,^[33]^ the co‐formation of Co(OH)_2_ is unavoidable during the CoCO_3_ precipitation even in saturated media and high pH. However, it's worth highlighting that this fact is not an issue for the developed process as the final product could be used as Li‐ion active material precursor and will be thermally treated.

**Figure 8:**
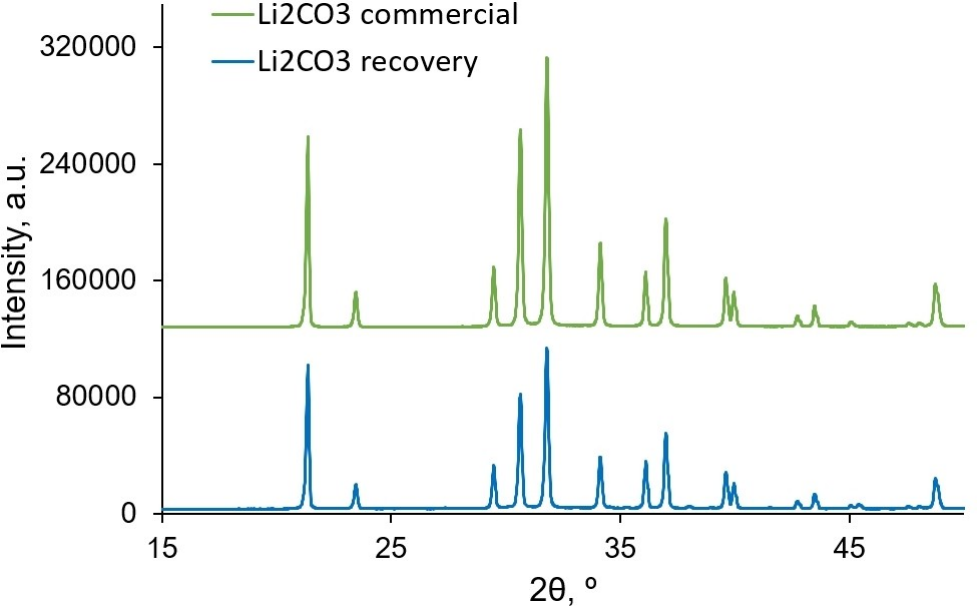
XRD patterns of Li_2_CO_3_ commercial powder (green curve) and XRD spectra of recycled Li_2_CO_3_ at the end of the recycling process (blue curve). Focus on the area of interest at theta ranging from 15 to 50°.

As mentioned previously, the water is added both by the addition of a small amount of HCl in the dissolution step and the Li_2_CO_3_ solution used in the precipitation step. Thus, there is a risk of progressive dilution of the DES with each additional re‐use cycle. That is why the water evaporation step is added before closing the recovery loop. The water evaporation is carried out in a rotary evaporator over 2 h under vacuum and heating to 50 °C, and it was verified that the condensate is only composed of water (Figure S6). A white precipitate appeared in the DES after the water was removed (shown in Figure 6 ‐ photo on the right) and was recovered by filtration. The residual Li concentration lower than 1 mg⋅L^−1^ was determined in the final DES filtrate after the cobalt recovery and the water evaporation. More than 99 % of the lithium originated both from the LCO material and the added Li_2_CO_3_ has thus been recovered. The precipitate was composed of LiCl and Li_2_CO_3_. However, as LiCl is much more soluble than Li_2_CO_3_, it was completely removed from the precipitate during the precipitate washing, the reason behind its non‐detection by XRD analysis of the final product, identified as pure Li_2_CO_3_ as shown in Figure 8, where the obtained diffractogram is compared to a diffractogram of a commercial Li_2_CO_3_ powder. The lithium presence in the filtrate was verified by AAS and the presence of chloride ions has been evidenced by the AgNO_3_ titration; the appearance of a grey precipitate of AgCl after AgNO_3_ addition proved the presence of chloride.

**Figure 9:**
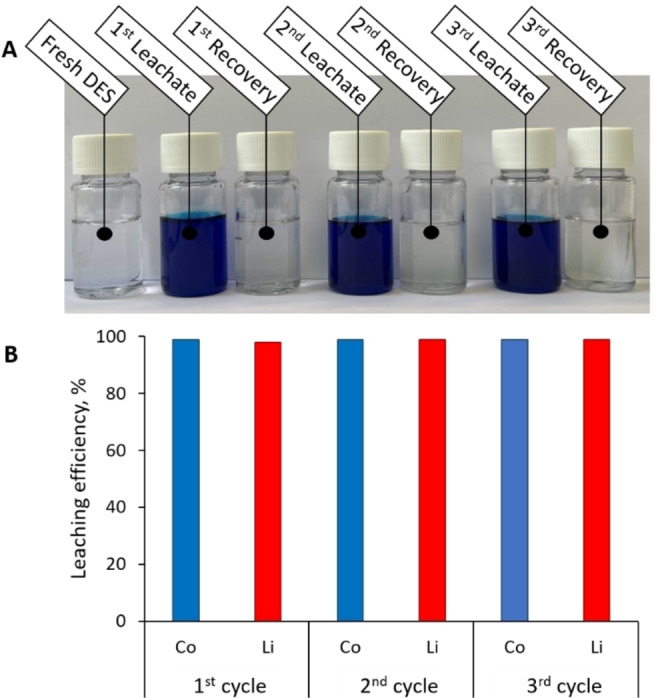
(A) Comparison a DES before and after each leaching and recovery cycle and (B) influence of number of reuse cycles on Li and Co leaching efficiencies (according to AAS determinations).

Finally, we wanted to verify the ability of the recovered DES to be reused for further leaching and recovery cycles. The DES was re‐used in three successive recovery cycles as depicted in Figure 9, while only the additional quantity of protons was added to the system at each re‐use cycle. Interestingly, no decrease in dissolution efficiency has been recorded over the performed dissolution/recovery cycles as shown in Figure 9, B. In addition, no color change is observed as shown in Figure 9, A.

Surprisingly, the majority of articles dealing with the application of DES in LCO leaching do not mention the reusability of the DES. To the best of our knowledge, there are only very few articles proving the good recyclability of DESs, highlighting the associated difficulties. Luo et al.^[34]^ observed the blue coloration of DES after the first recovery cycle, as well as the loss of one of the two components of the used DES. While Morina et al.^[12]^ have carried out four successive cycles of metal (Co, Ni) recovery, they have evidenced the progressive accumulation of Li within the system and a decrease of leaching efficiency at the 4^th^ cycle. Moreover, the authors did not examine the degradation of the DES system based on ChCl:lactic acid, prone to degradations as described by Rodriguez‐Rodriguez et al.^[24]^ In our case, the DES recovered after cobalt and lithium precipitation and water evaporation remains colorless, testifying to the stability of its composition. Furthermore, no decrease in dissolution efficiency has been observed over the three successive leaching‐recovery cycles, which is a very positive result compared to the literature.

**Figure 10:**
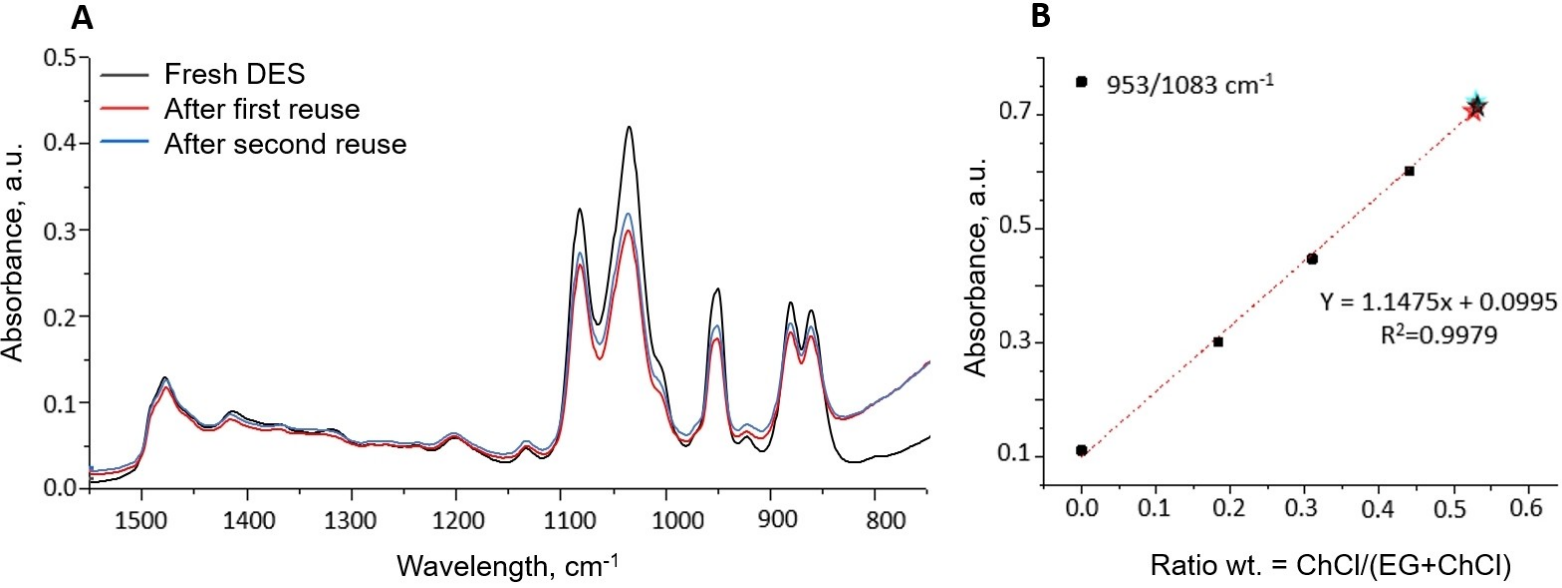
Influence of the number of reuse cycles of DES on its composition examined by FTIR spectroscopy (700–1550 cm^−1^). (A) FTIR spectrum obtained for a fresh DES (A, black curve), after the first reuse cycle (A, red curve), after the second reuse cycle (A, blue curve). (B) Absorbance ratio of 953/1083 cm^−1^ signals as a function of ChCl/(EG+ChCl) ratio. The absorbance ratios corresponding to the fresh and used DES obtained from their FTIR spectra are reported on the curve by the stars corresponding to fresh DES solvent (B, black star), solvent after the first reuse cycle (B, red star), solvent after the second reuse cycle (B, blue star).

To confirm that the DES can be reused and that its composition remains unchanged over the reuse cycles, an FTIR analysis has been performed by comparing the IR spectra of the initial and the used DESs. FTIR spectroscopic analysis (Figure 10, A) performed on the fresh DES, after the first and after the second reuse cycle, showed similar spectral signatures, namely indicating the presence of bands characteristic of ammonium at 953 cm^−1^, 1133 cm^−1^ and 1479 cm^−1^ and the presence of a peak characteristic of the C−O bond of aliphatic ethers at 3050 and 1700 cm^−1^ (not shown), both present in EG and in ChCl, which supports our assumption that the DES has not been degraded or that the degradation leads to volatile species, which cannot be excluded. Moreover, it is useful to highlight that the signs of degradation observed by Schiavi et al.^[22]^ in FTIR spectra in the case of a thermally treated ChCl:EG mixture (72 h, 180 °C) have not been detected in our case. It is worth to note that quite harsh ageing conditions have been used by these authors.

Several authors have proposed the preferential degradation of one of the components related to either its thermally degradation or/and the degradation induced by Co reduction and leaching.[^19^, ^22^, ^34^] In order to verify the variation of ChCl:EG ratio in the studied system, several solutions containing different ratios of ChCl in EG have been prepared and analyzed by FTIR spectroscopy. A reference curve (Figure 10, B) was then plotted, displaying the variation of the absorbance ratio of the absorbance bands at 953 cm^−1^ and 1083 cm^−1^, corresponding to the functional groups belonging to choline chloride and to ethylene glycol, respectively, as a function of ChCl/(EG+ChCl) mass ratio. The ratio of the absorbance bands at 953 cm^−1^ and 1083 cm^−1^ obtained for the fresh DES, after the first and the second reuse cycle have been represented by a black star, a red star and a blue star, respectively, in the plot (Figure 10, B). Their perfect overlay with the starting ChCl:EG mass ratio of 0.55 unambiguously shows that the DES is not subject to any degradation during the developed process or that the degradation is very limited and difficult to detect, meaning that the DES could likely be further (re−)used.

These results are confirmed by NMR analysis performed on the DES recovered after water evaporation. The obtained spectrum (shown in Figure S9) exhibits the same signatures as the initial DES and no degradation products are detected. Moreover, proton signals obtained after the recovery process do not evolve, as shown in Figure S9; the signals at 2.7 ppm and 3.5 ppm are associated with three CH_3_ groups (9 protons) of the ammonium and one CH_2_ group of OH−CH_2_ (2 protons) of ethylene glycol, respectively. The signal at 3 ppm is associated with one CH_2_ group (2 protons) from the ammonium and two CH_2_ groups of the glycol (8 protons considering the 1 : 2 ratio of ChCl:EG). In order to check for a possible depletion or accumulation of chloride ions within the DES system, the quantity of chloride remaining in the DES after the 3^rd^ cycle was determined. A chloride titration with AgNO_3_ solution, followed by a potentiometric titration step, was carried out (not shown), confirming that the successive leaching and precipitation steps had no consequence for the Cl^−^ concentration within the DES. This constant concentration allowed to confirm that there is no significant chloride depletion or accumulation; thus, the DES can even be reused in further leaching steps.

## Conclusions

The hydrometallurgical processes currently used for lithium‐ion battery recycling generally focus on the use of acid for the leaching step, providing the required high leaching efficiency for the targeted metals (cobalt, lithium…). More recently, the use of alternative leaching media, such as DESs, has attracted the attention of researchers. However, no solution is perfect, and it was shown that good leaching performance cannot be achieved without a degradation of the DESs, thus compromising their reuse.

In this context, our study proposed to associate a deep eutectic solvent (DES) based on choline chloride and ethylene glycol to a small additional amount of hydrochloric acid to reach a quantitative dissolution of the LCO active material. It was demonstrated that the acidity of the medium plays a key role in the dissolution efficiency, whereas the DES contributes to an improvement in the Co extraction and complexation as judged by the low leaching efficiency determined in an acidic aqueous medium of equivalent proton concentration. Thus, the quantity of acid to be added to the DES is fully dependent on the cobalt quantity to be dissolved. It was determined that more than 3 protons must be added per one atom of cobalt to obtain a complete dissolution of the metal. Moreover, under these experimental conditions, the quantitative LCO dissolution has been reached at shorter times and lower temperatures compared to the literature, while no DES degradation has been detected during leaching, precipitation or the water evaporation steps. It is important to note that, at the end of the recovery process, only DES and water remain in the resulting solution. Considering these advantages, a further up‐scaling of the process might be interesting to verify the feasibility of an industrial application. In parallel, it has to be outlined that the DES can be recycled and reused for further leaching operations, significantly reducing the environmental impact of this recovery process.

For our future work, testing the robustness of the process and its ability to dissolve other and more complex types of active materials would be of primary importance. The ability of the recovered material to be reused as an active material precursor for Li‐ion batteries is also interesting for our future work.

## Materials and Methods

### Materials

Two types of LCO samples have been used in this work, namely a commercial lithium cobalt oxide (LiCoO_2_) powder supplied by Sigma‐Aldrich and a lithium cobalt oxide cathode obtained from a spent drone lithium‐ion battery of EPS brand composed of an aluminum current collector, a binder, carbon particles and an LCO active material. The waste LIB cathodes have been obtained after a careful opening of the spent LIBs in a glove box under argon atmosphere. Prior to the opening, the LIBs have been properly discharged.^[35]^ After the opening, the anodes, the cathodes and the separator have been manually separated and only the cathodes have been used in this study. The characterization of the cathode material shows a specific capacity of 135 mAh⋅g^−1^ (loading of 0.022 g⋅cm^−2^ of LCO). The choline chloride (purity of 99 %) and ethylene glycol (purity of 99 %) have been supplied by Sigma Aldrich and have been used as received. Hydrochloric acid, HCl (ACS reagent, 8 mol⋅L^−1^), and lithium carbonate, Li_2_CO_3_ (ACS reagent, ≥99.0 %), have been purchased from Sigma‐Aldrich.

### Leaching experiments

Prior to launching the leaching experiments, the DES has been prepared by mixing choline chloride and ethylene glycol with molar ratio of 1 : 2, respectively, at 50 °C during 20 min. Fresh DES has always been prepared just prior to the experiments with the exception of the experiment examining the DES reusability. At first, the leaching efficiency of a pure ChCl:EG system was compared to a system with additional HCl at a fixed powder/DES wt. ratio (1/50), constant temperature of 87.5 °C (chosen arbitrarily) and time (2 h). Typically, a mass of 1 g of LCO was dissolved in 50 mL of DES. Different H^+^/Co ratios (n(Co)=x⋅n(H^+^)) have been tested ranging from 0 to 6, where a small quantity of a concentrated HCl (8 M) has been added to the pure DES in order to achieve the selected H^+^/Co ratio. As an example, in the case of H^+^/Co ratio equal to 3, 1 g of LCO was dissolved in 50 mL of DES previously acidified with 5 mL of 8 M HCl, thus attaining the final HCl concentration of 0.8 mol⋅L^−1^.

Once the optimum quantity of protons had been determined, the influence of the reaction temperature was explored between 40–87.5 °C while keeping the DES/LCO wt. ratio, time and HCl concentration fixed. Furthermore, the dissolution kinetics were followed at the previously selected optimum conditions. All experiments have been carried out both with the commercial LCO powder and the spent cathodes. In addition, these results have been compared to results obtained in HCl aqueous medium of equivalent proton concentration.

### Recovery experiments

In order to be able to reuse the DES system in further leaching cycles, it was necessary to find a way to circumvent DES degradation during recovery of the dissolved elements. Precipitation by either the addition of lithium carbonate or lithium hydroxide has been tested at two different pH values (pH 10 and 12). A cobalt carbonate/hydroxide precipitate was obtained at room temperature by adding lithium carbonate/lithium hydroxide solution dropwise (0.2 mol⋅L^−1^) up to reaching the desired pH value (monitored using a pH meter) and filtered. However, as explained in the Results and Discussion section, the addition of lithium carbonate was found preferrable over lithium hydroxide addition. The lithium contained in the DES was followingly precipitated by water evaporation from the DES. After filtration, the final Co and Li concentrations in the DES were determined and the precipitation efficiency of cobalt and lithium was calculated.

### DES reuse

Prior to DES reuse, the additional water contained in the DES due to the acid addition and Co and Li precipitation (addition of 0.2 M Li_2_CO_3_) was removed using a rotary evaporator over 2 h under vacuum and heating to 50 °C, leading to the lithium carbonate precipitation as previously explained. The recovered DES was analyzed by FTIR and NMR spectroscopy in order to check its state and was followingly used for two more successive leaching and precipitation cycles. The obtained condensate was also analyzed in order to determine its composition. Prior to launching a new leaching cycle, the water was always removed by evaporation.

The leaching experiments have been carried out at the optimum leaching conditions determined previously. The precipitation has been achieved by lithium carbonate solution addition up to reaching the desired pH as described previously. Both leaching and precipitation efficiencies have been systematically determined for each cycle and the state of the recovered DES was always verified.

### Characterization methods

#### Concentration and leaching efficiency determination

The concentration of Co and Li in the DES have been determined using a PinAAcle 900F, Perkin Elmer® atomic absorption spectrometer, equipped to work both in emission and absorption mode using an acetylene/air flame (ratio of 2.5/10 vol/vol). The Co concentration has been determined in the absorption mode using a Co lamp at the wavelength of 240.73 nm. The Li concentration has been determined using the emission mode at the wavelength of 760.78 nm. The accuracy of the apparatus is assumed to be within a ±0.5 % range. The concentration determinations have been based on calibration curves (ranging from 1 to 20 mg⋅L^−1^ for Co and from 0.2 to 10 mg⋅L^−1^ for Li); the standards have been prepared by dilution of a stock solution of 1000 mg⋅L^−1^ from ROTI®Star. The DES solutions, being quite concentrated, have been systematically diluted with pure water prior to the analyses in order to be within the calibration range. Finally, the leaching efficiency was defined as follows (Equation 5:

(5) $$\eta_{i}=\;\frac{\left[M_{i}\right]\times \;V_{sol}}{m_{i}\;}\times 100$$

Where [M_i_] corresponds to metals’ concentration (Li or Co) in solution based on the AAS analysis and the applied dilution factor (mg⋅L^−1^). V_sol_ is the DES total volume (L) and m_i_ is the metal (Co or Li) mass (mg) contained in the solid introduced in the DES.

#### Cobalt speciation determination

Metal‐containing leachates were also analyzed using an Ultraviolet‐visible (UV‐Vis) spectrophotometer, Varian Cary® 50. Several optical paths have been used depending on the concentration of the solutions (using quartz cells of 0.1 mm). These analyses allowed us to determine the cobalt speciation in DES solution using photons absorbed by the analyzed solution in a wavelength range from 200 to 800 nm. All determinations have been carried out against the DES blank, and no dilution has been carried out.

A preliminary UV‐vis spectroscopy measurement allowed us to calculate a molar extinction coefficient (ϵ) of 550 L⋅mol^−1^⋅cm^−1^ for the formation of the cobalt tetrachloride complex in an acidic medium (Figure S7) and a molar extinction coefficient (ϵ) of 609 L⋅mol^−1^⋅cm^−1^ for the formation of the cobalt tetrachloride complex (CoCl_4_^2−^) in the medium proposed in this study (Figure S8). These values are in agreement with those reported in the literature^[27]^ and have been used to determine CoCl_4_^2−^ concentration in this study.

#### DESs characterization

Deep eutectic solvents were analyzed by Fourier transform infrared (FTIR) spectroscopy using a Vertex 70 V FTIR apparatus equipped with a diamond ATR module (operating wavelength: 500−4000 cm^−1^ range). This analysis allowed us to check the state of degradation of the DES solvent before and after the leaching process.

The solutions of DES were also analyzed by Nuclear Magnetic Resonance (^1^H NMR) spectroscopy using an NMR 400 MHz, BRUKER, Avance III HD spectrometer, which allows the identification of the nature and the proportion of the DESs components (choline chloride/ethylene glycol) and thus the DES degradation. For each solution, an insert composed of 0.1 mol⋅L^−1^ of tetraethylammonium tetrafluoroborate (TEAB) in DMSO‐d_6_ was immersed in the solution contained in the NMR tube.

#### Characterization of solids

The surface morphology and the chemical composition of the solid materials (the cathode material, the commercial powder and the leaching residues) have been determined by Scanning Electron Microscopy (SEM) using a MEB FEG ZEISS Ultra 55, in both secondary electron and retro diffusion electron mode. EDX analyses were systematically coupled to SEM observations. In addition, precipitates have been analyzed by X‐ray diffraction using an X′Pert Pro MPD diffractometer from PANalytical, and by XPS, using a Versaprobe II ULVAC‐PHI spectrometer. The deconvolution of the XPS data peaks has been performed using the ThermoScientific ^TM^ Avantage data system ^TM^ software.

## Supporting Information

The authors have cited additional references within the Supporting Information.[^16^, ^20^, ^23^, ^28^, ^36^, ^37^, ^38^, ^39^, ^40^, ^41^, ^42^, ^43^, ^44^] The Supporting Information contains a non‐exhaustive list of acid leaching agents, the physico‐chemical properties of some candidate DESs, a comparison of ^1^H NMR spectra of the fresh and the aged DES, a comparison of UV spectra of the fresh and the aged DES, a FTIR spectrum of water evaporated from the DES, UV spectra of cobalt(II) complexes in the ChCl:EG DES and HCl media, SEM photos coupled with EDS analyses of the leaching residues and the equation of the choline chloride Hoffman degradation mechanism.

## Conflict of interest

The authors declare no conflict of interest. The funders had no role in the design of the study; in the collection, analyses, or interpretation of data; in the writing of the manuscript, and in the decision to publish the results.

1

## Supporting information

As a service to our authors and readers, this journal provides supporting information supplied by the authors. Such materials are peer reviewed and may be re‐organized for online delivery, but are not copy‐edited or typeset. Technical support issues arising from supporting information (other than missing files) should be addressed to the authors.

Supporting Information

## Data Availability

The data that support the findings of this study are available from the corresponding author upon reasonable request.
