# Supplementary material for: Lithium‐Ion Battery Cathode Recycling through a Closed‐Loop Process Using a Choline Chloride‐Ethylene Glycol‐Based Deep‐Eutectic Solvent in the Presence of Acid
Source: ChemistryOpen. 2023 Jul 26;13(2):e202300061. doi: 10.1002/open.202300061 (PMC10853073; doi:10.1002/open.202300061)
Supplement: Supplementary file 1 — Supporting Information [file OPEN-13-e202300061-s001.pdf]

ChemistryOpen

Supporting Information

Lithium-Ion Battery Cathode Recycling through a Closed-Loop Process Using a Choline Chloride-Ethylene Glycol-Based Deep-Eutectic Solvent in the Presence of Acid

Delphine Yetim, Lenka Svecova,* and Jean-Claude Leprêtre

Table S 1 Non-exhaustive list of acidic leaching agents used in the literature with the corresponding experimental conditions and the achieved leaching efficiencies.

Cathode active material	Leaching agent	Temperature (°C)	Efficiency (%)	Time (h)	Ref.
$LiCoO_2$	3 M H_2SO_4	70	Li=98, Co=98	6	[36]
	2 M H_2SO_4 + 6% v/v H_2O_2	60	Co=98, Li=99	1	[39]
	4–8% H_2SO_4 + 2-4 % H_2O_2	50 – 80	Co=30	1	[37]
	3 M H_2SO_4 + 3% H_2O_2	70	Co=100, Li=100	7	[36]
	4 M HCl	80	Co=99, Li=99	1	[38,39]
	1.25 M Citric acid + 1% H_2O_2	90	Co>90, Li=100	0.5	[16]
$LiMnO_2$	2 M HNO_3	80	Mn=95, Li=100	2	[40]
NMC (811)	4 M HCl	25	Co=99, Mn=99, Ni=99, Li=99	4	[28]
$LiMnO_2$, $LiCoO_2$, $LiNi_{1/3}Mn_{1/3}Co_{1/3}O_2$	4 M HCl	80	Co=99, Mn=99, Ni=99, Li=99	1	[23]
LNCA	4 M HCl	90	Li, Ni, Co, Al >80	1	[41]

Table S 2 Physico-chemical properties of some of candidate DESs.

HBA	HBD	Molar ratio	Density ($g.cm^{-3}$)	Viscosity (mPas·s)	Conductivity ($mS{\cdot}cm^{-1}$)	Ref.
ChCl	EG	1 : 2	1.2	36-52 (25 °C)	7.61	[42]
ChCl	Oxalic acid	1 : 1	1.28	126 (28 °C)	-	[43]
ChCl	Malonic acid	1 : 1	1.29	1000 (30 °C)	>0.5*	[43]
				494 (80 °C)	-	[44]*
ChCl	Urea	1 : 2	1.25	1571 (25 °C)	-	[20]

**Conductivity measured at 30 °C for a viscosity of 1000 mPas·s.*

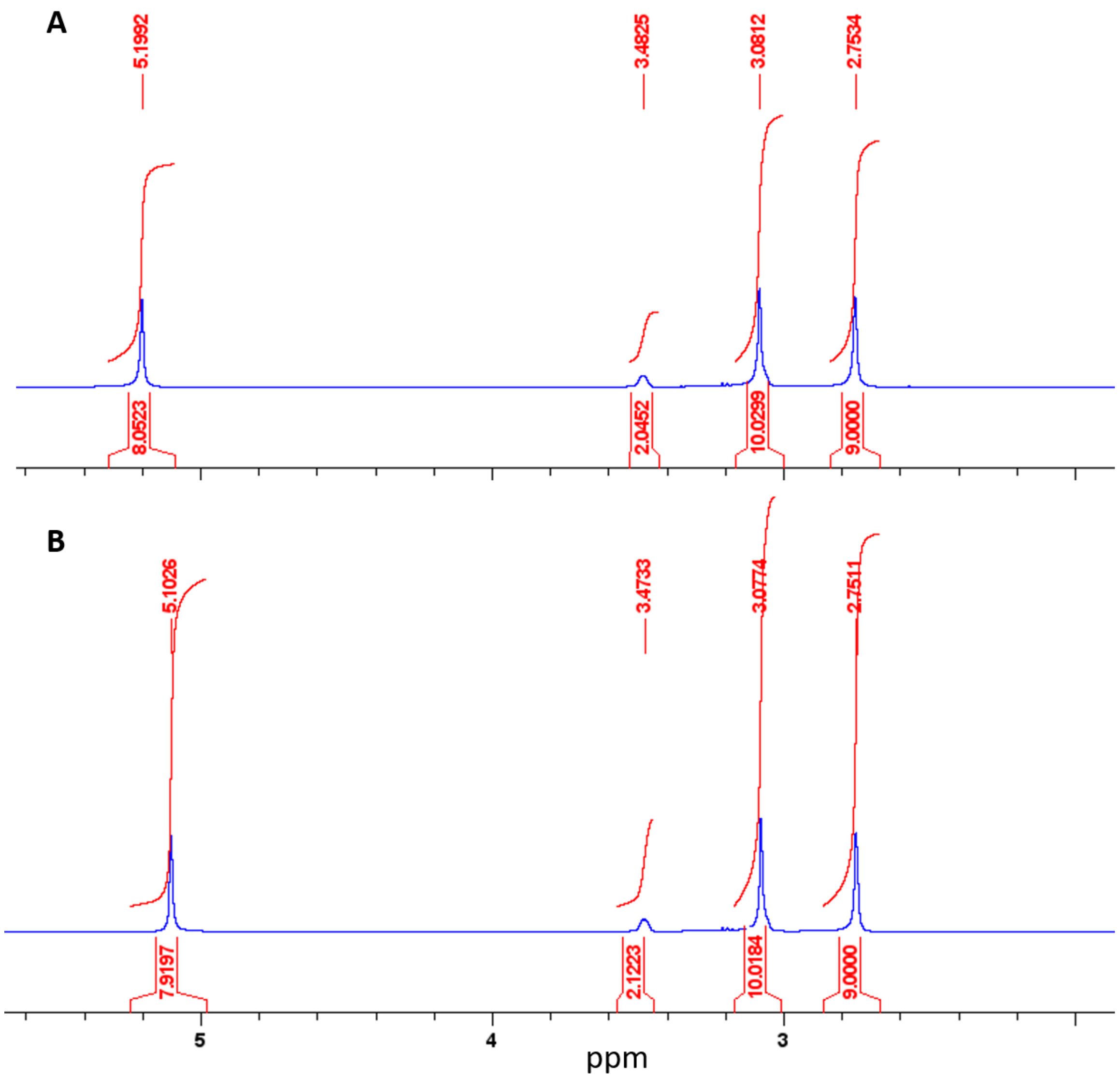

Figure S 1 ¹H NMR of the DES mixture with 10% wt. of HCl after preparation (A) and after heating at 87.5°C during 2 hours (B).

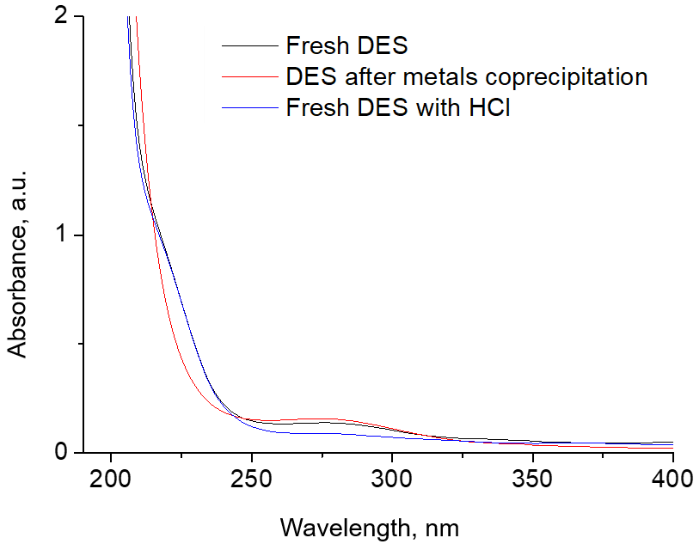

Figure S 2 Visible UV spectra of fresh DES with and without HCl and after metal precipitation.

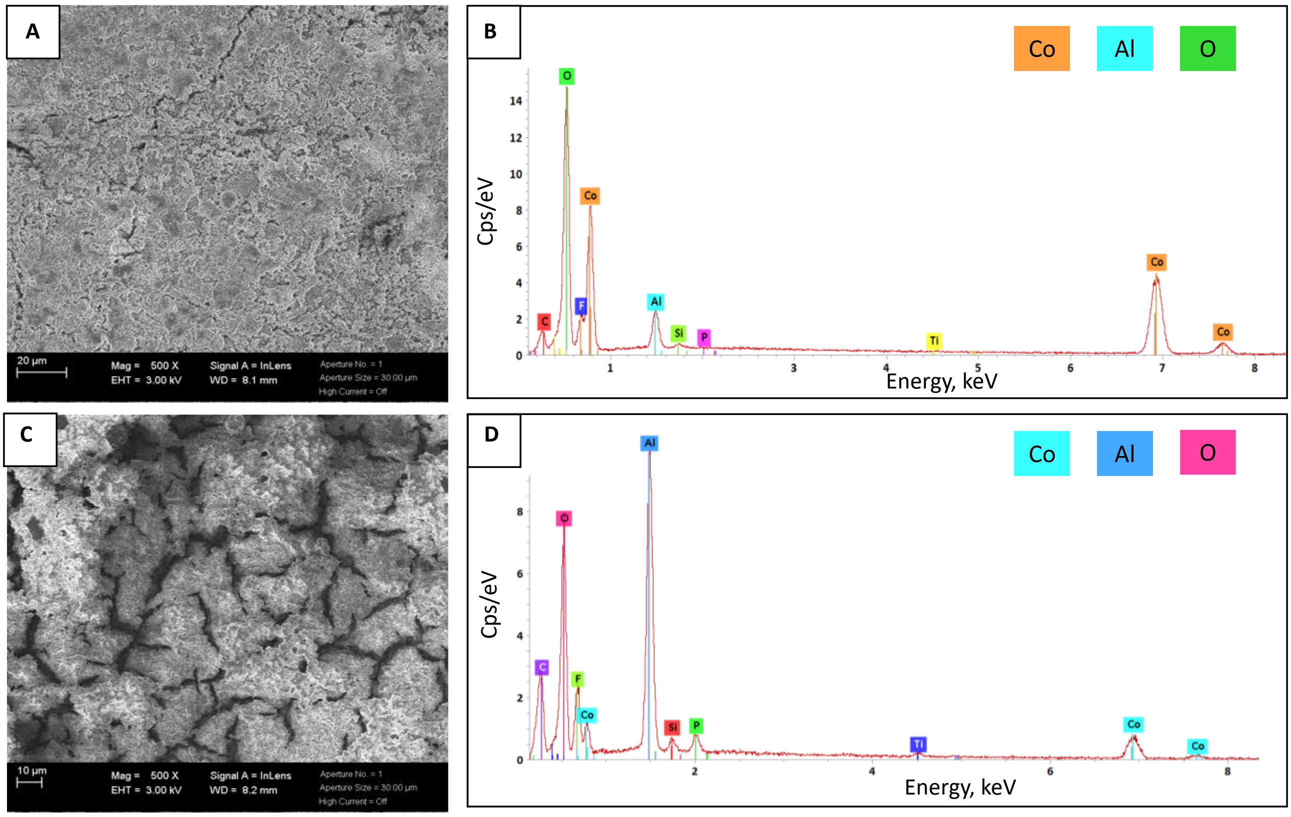

Figure S 3 Scanning electron microscopy images of spent cathode, after 10 min dissolution x500 zoom (A), and after 40 min dissolution x500 zoom (C), and EDS elemental analysis after 10 min (B) and 40 min (D).

After 10 min (Figure S3, A), the SEM analysis shows the presence of small cracked areas appearing on the initial cathode material. These zones are covered with small grains, which are composed mainly of cobalt oxide on the surface according EDS analysis (Figure S3, B).

After 40 min, a large network of cracked zones is visible in the sample (Figure S3, C). The areas composed of structures in the shape of rose petals are increasingly dense and sprinkled with small grains of about 55 nm, which were identified as carbon particles that ensure the cathode conductivity. The EDS analysis after 40min of leaching (Figure S3, D) shows the progressive disappearance of the Co signature whereas the Al oxide presence was mainly detected.

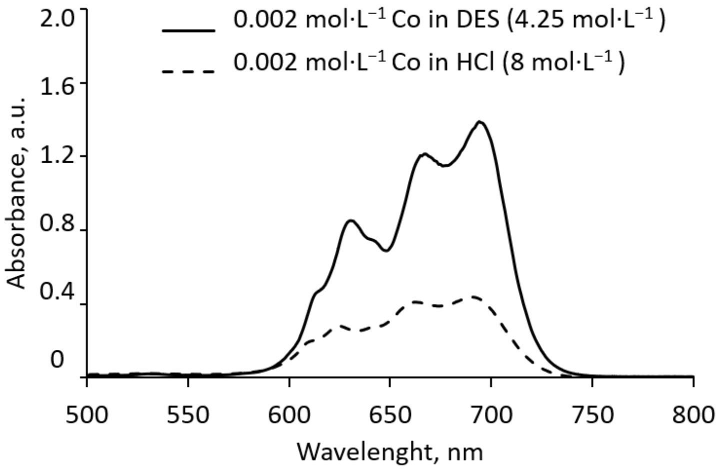

Figure S 4 Visible UV spectra comparison of solution containing 0.002 mol.L-1 of Co dissolve in DES (4.25 M of chloride) and in HCl (8M of chloride) media.

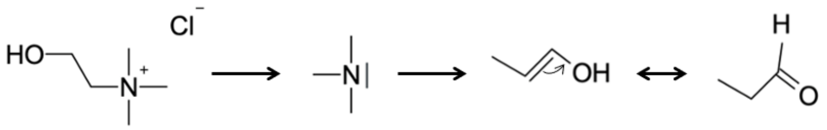

Figure S 5 Choline chloride Hoffman degradation.

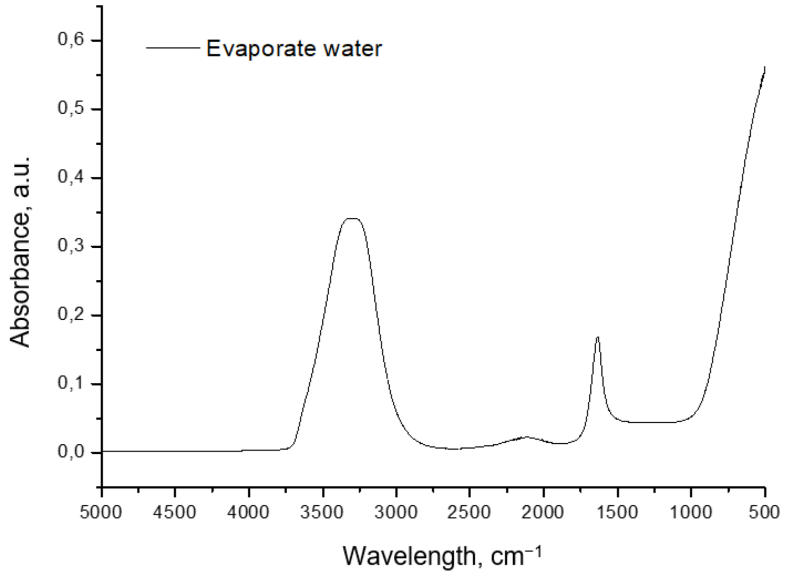

Figure S 6 FTIR spectroscopy (700 cm^{-1}- 1550 cm^{-1}) of evaporated and condensed water obtained from rotary evaporator.

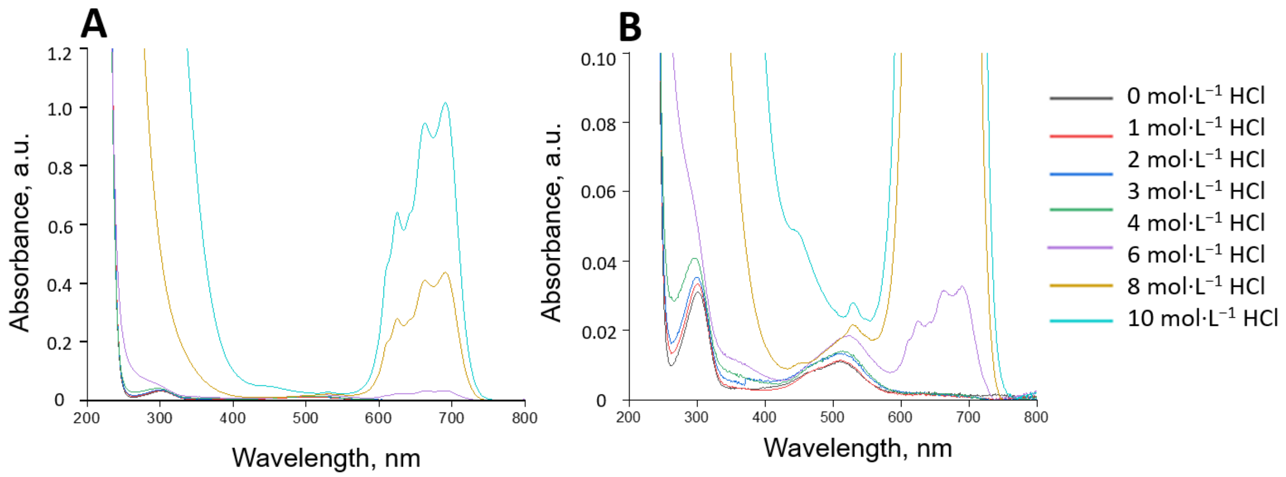

Figure S 7 Visible UV spectra of cobalt (II) complex formation in different concentration of HCl from 0 to 10 mol·L^{-1} (A), and zoom at Abs bellow 0.10 (B). A concentration of 0.002 mol·L^{-1} Co has been prepared in each solvent for these experiments.

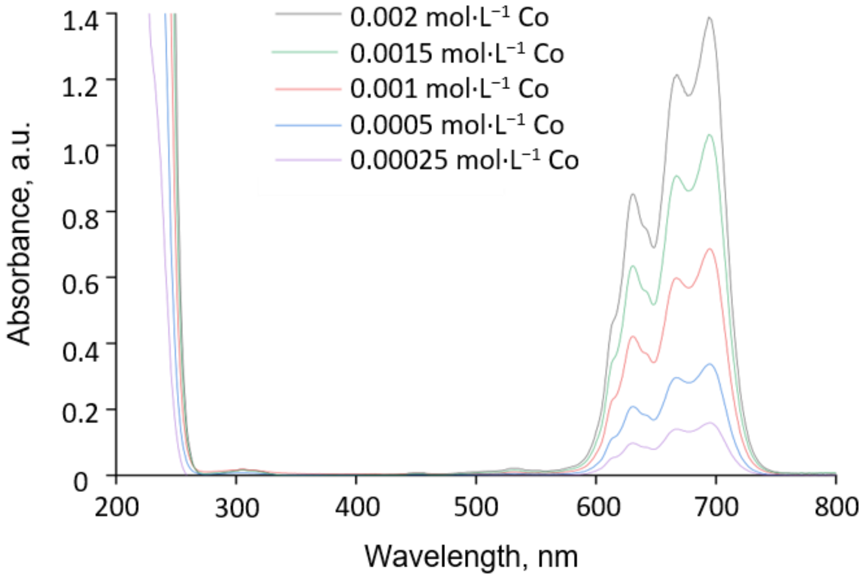

Figure S 8 UV-Visible spectra of cobalt (II) complex formation for different concentrations of cobalt from 0.00025 to 0.002 mol·L^{-1} in ChCl:EG (1:2).

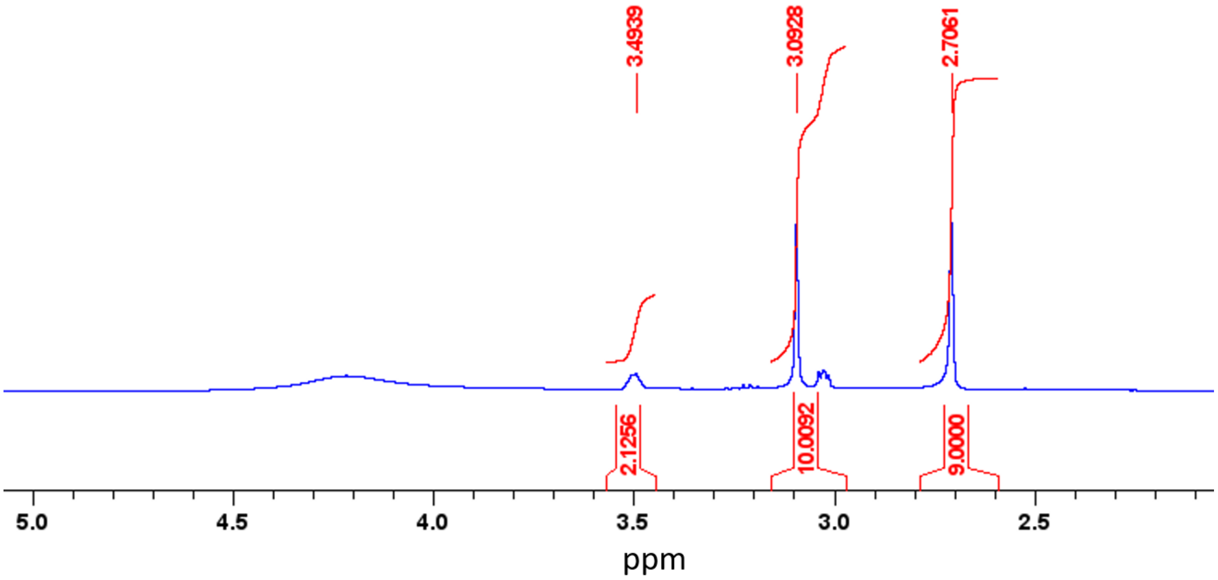

Figure S 9 ^{1}H NMR of the DES that has been submitted to leaching and precipitation steps and finally recovered after water evaporation.
